# Advances in Wire EDM Technology for Cutting Silicon Carbide Ceramics: A Review

**DOI:** 10.3390/ma18173955

**Published:** 2025-08-23

**Authors:** Mohammad Ghasemian Fard, Jana Petru, Sergej Hloch

**Affiliations:** Faculty of Mechanical Engineering, VSB—Technical University of Ostrava, Poruba, 70800 Ostrava, Czech Republic; m.ghasemian3058@gmail.com (M.G.F.); jana.petru@vsb.cz (J.P.)

**Keywords:** silicon carbide, wire electrical discharge machining, micro-cracks

## Abstract

Silicon carbide (SiC) ceramics have gained significant attention in advanced engineering applications because of their superior mechanical properties, resistance to wear and corrosion, and thermal stability. However, the precision machining of these materials is extremely challenging because of their intrinsic hardness and brittleness. Wire Electrical Discharge Machining (WEDM) has become increasingly popular as a viable technique for processing SiC ceramics because of its ability to produce intricate geometries and high-quality surface finishes. In this review paper, a comprehensive overview of WEDM technology applied to SiC ceramics is presented, emphasizing the influence of process parameters, wire materials, and dielectric fluids on cutting efficiency and quality. This research explores recent experimental findings related to Wire Electrical Discharge Machining (WEDM) and highlights the challenges in reducing material damage. It also presents strategies to improve machining performance. Additionally, potential future directions are discussed, providing a roadmap for further research and the application of WEDM in processing silicon carbide (SiC) and its variants, including solid silicon carbide (SSiC) and silicon-infiltrated silicon carbide (SiSiC).

## 1. Introduction

Silicon carbide (SiC) ceramics are highly valued for their exceptional hardness, thermal stability, and resistance to wear and corrosion, making them indispensable in various high-performance applications. However, these same properties present significant challenges in machining and disintegration processes. Traditional methods are not suitable and often fail, and some advanced manufacturing methods even fail to achieve the precision and surface quality required for SiC ceramics (SiC). SiC ceramics are used in various high-performance applications because of their special mechanical properties, listed in Table 1. The semiconductor industry has further fueled this interest as it requires state-of-the-art materials that can efficiently function in high temperatures and high-power environments. The melting point of SiC is approximately 2730 °C, making it suitable for applications where superalloys are typically used. The strength, fracture toughness, and hardness of forged SiC are generally higher than those of hot-pressed SiC [1].

Demand for silicon carbide (SiC) as a semiconductor due to its wide range of operating temperatures and energy efficiency has led to sustainable growth [16]. With an energy gap of 3.32 eV, SiC has a breakdown strength of 3 MV, which is 10 times that of Si because SiC’s energy gap is more than three times that of Si [17,18,19]. With this property, the use of these semiconductor devices is possible in a variety of industries from marine to aerospace. The machining of silicon carbide (SiC) ceramics, as a natural hard, brittle material, is challenging due to their high hardness, strength, low fracture toughness, and brittleness [20,21,22,23]. The primary drawbacks of ceramics are their cost and the complexity of their manufacturing process, particularly in the finishing stage. When it comes to the traditional machining of hard ceramic structures, the main limitation is the inability to achieve net-shape fabrication. As a result, there is a need for the additional machining of ceramics, which often involves a low material removal rate (MRR). Remarkable progress has been made in the study of machining SiC ceramics; nevertheless, research to accurately determine the characteristics of surface deformation and material removal mechanisms in these materials is still ongoing. It is essential to understand the material removal mechanisms in order to achieve accurate and effective SiC ceramic machining.

In this review, we summarize the current understanding of WEDM for SiC and analyze recent developments. The combination of adaptive control systems and advanced simulation techniques is a possible future direction to overcome the current limitations that is discussed in this review paper. The primary goal of this study is to fill research gaps in the understanding of WEDM’s potential and constraints, which will promote innovation and broader use in the machining of silicon carbide ceramics.

### 1.1. Machining Characteristics of Silicon Carbide Ceramics

Recent studies have focused more on cutting-edge materials such as silicon carbide (SiC) ceramics, which are renowned for their remarkable hardness, thermal stability, and corrosion and wear resistance. Silicon carbide ceramics are also well known for their extraordinary mechanical, thermal, and radiation hardness qualities, as well as optical and biocompatibility qualities. They are currently often used in many different applications, including turbines, bearings, the automotive industry [24], the aerospace industry [25], heat engine components, prosthetic bio-medical implants [26], and others. Machining silicon carbide (SiC) is an expensive, time-consuming and resource-intensive process, significantly limiting its widespread use in industrial applications. Given its challenging mechanical characteristics, SiC is categorized as a difficult-to-machine ceramic, and more economical and effective machining techniques are required [20,21,22,23]. Conventional grinding involves high temperatures and different forces; this frequently leads to surface cracks, severe subsurface damage, and rapid tool wear, which then lead to high manufacturing costs and low productivity. Moreover, SiC’s adaptability to a wide range of next-generation innovations is exemplified by its potential use in microelectromechanical systems (MEMSs), optical devices, radiation detectors, biomedical implants, high-temperature electronics, photovoltaic systems, and heat exchanger technologies [27,28,29,30]. These features make SiC ceramics an excellent choice for next-generation components in highly demanding applications. However, conventional machining methods such as turning, milling, and drilling are not often sufficient for effectively machining SiC ceramics due to their extreme hardness and brittleness [31]. A low rate of material removal, accelerated tool wear, and increased surface roughness are the results of the uneven distribution of reinforcing particles along with high hardness and brittleness [32]. In addition, the final stages of machining or grinding often result in defects in the surface of ceramic components. Achieving a high rate of material removal without sacrificing the surface quality of advanced ceramics is generally difficult. Accordingly, the machining of ceramics is usually very expensive, making up about 80 percent of the overall cost of manufacturing. Researchers are increasingly using cutting-edge machining techniques that are well-suited for processing composite materials to create complex shapes [33,34]. Table 2 provides an extensive summary of the essential characteristics of SiC that emphasize these machining challenges.

The Wire Electrical Discharge Machining technique has demonstrated notable potential for machining modern ceramics like silicon carbide (SiC), but critical challenges still remain [35,36]. There are several restrictions regarding how well WEDM can work with silicon carbide ceramics, even though it can precisely cut hard and brittle materials. Current investigations demonstrate that process parameters affect cutting efficiency, tool wear, and surface integrity. Microcracks and thermal distortions are examples of surface damage that continue to be a serious problem and impair the quality and functionality of SiC components. The adoption of WEDM in industrial applications is further complicated by the absence of standardized techniques for parameter optimization and real-time monitoring systems. Even with improvements in wire materials, dielectric fluid composition, and hybrid machining methods, it is still unknown how well they balance the rate of material removal with surface quality. To determine how particular issues like wire breakage, tool wear, and heat-affected zones affect the machinability of silicon carbide ceramics, more investigations are required.

### 1.2. SiC Ceramic Types: Implications for WEDM Performance

SiC ceramics come in several forms, each with different purities, microstructures, and electrical properties, which in turn affect their machinability in WEDM applications. For example, SSIC is an extremely hard and strong monolithic form of silicon known for its long life, hardness, and strength, but its low electrical conductivity makes it hard to machine unless conductive coatings or additives are applied. By contrast, silicon-infiltrated silicon carbide (SiSiC) holds free silicon, improving electrical conductivity, which makes it more suitable for the direct WEDM process without the need for any additional conductive layers. Conductive doped SiC is another important form, in which SiC is doped with elements such as boron, nitrogen, or aluminum to improve its electrical conductivity. Each SiC type offers different trade-offs in terms of machinability, thermal response, and surface integrity outcomes, necessitating precise selection based on the specific engineering application. These variants are frequently used in high-performance applications like semiconductors, where both electrical and mechanical performance are critical. It is necessary to comprehend these distinctions in order to optimize WEDM parameters and ensure effective material removal while minimizing surface defects. Table 3 lists each variant’s relevant characteristics and WEDM suitability to give a better idea of how they compare.

### 1.3. Fundamentals of Wire Electrical Discharge Machining

Material erosion using controlled electrical sparks, which forms the basis of modern EDM, was first investigated in the 1940s and has since been refined into the highly precise machining process it is today.

**Figure 1:**
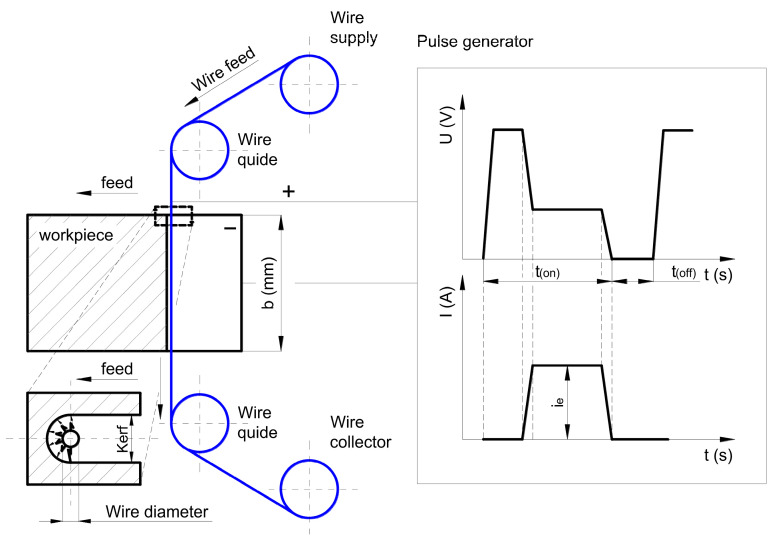
Schematic of Wire Electrical Discharge Machining (WEDM).

Nowadays, we are witnessing an increasing focus on cutting-edge machining methods to achieve higher precision levels that meet the complexity requirements for modern industries. The unconventional, extremely accurate machining technique known as Wire Electrical Discharge Machining is frequently used in sectors where precision and the capacity to produce complex parts are essential. This technique requires several key resources: an electrically charged, continuously moving wire (the electrode); a dielectric fluid that acts as an insulator and flushing agent; and a power supply (pulse generator) to create discharges. These components are used to cut complicated shapes into electrically conductive materials such as metals, ceramics, and composites. Crucially, WEDM has grown in popularity due to its adaptability and affordability, particularly in industries where strict quality standards are required, such as electronics, automotive, aerospace, and medicine industries [37,38]. A schematic illustrating the operation of this method is presented in Figure 1. A critical factor enabling the use of SiC ceramics in Wire Electrical Discharge Machining (WEDM) is their electrical conductivity.

WEDM technology provides incredible precision in the creation of intricate geometries and complex shapes, which is hard to achieve with conventional methods [39]. In contrast to traditional machining, the non-contact nature of WEDM allows it to process materials of varying hardness without inducing mechanical deformation or distortion, as the tool does not make direct contact with the workpiece. This characteristic is beneficial for machining in delicate sections, especially for hard components, such as punches, dies, and thick metal plates, while maintaining surface quality [40]. Moreover, WEDM produces minimal burrs and achieves high-quality surface finishes, minimizing the reliance on post-processing and improving overall efficiency. The main privilege of this method is its ability to machine fine features with reduced distortion, ensuring dimensional accuracy and overall integrity of the parts [41]. Over time, this technology has substantially moved forward, extending its uses beyond the shaping of conductive hard materials to the machining of low-conductivity materials such as composites and ceramics [38]. This approach is particularly helpful for components that are difficult to machine using conventional techniques. Wire EDM is a highly accurate, non-contact procedure for machining materials by melting and evaporating, therefore preventing mechanical stress [42]. Si and Ge are examples of hard, brittle materials that can be effectively cut using this technique [43]. It can also be used to slice a variety of conducting materials with better mechanical properties and higher efficiency, such as superalloys [44,45,46]. Additionally, the use of extremely thin cutting wires allows for achieving an exceptionally sharp corner radius [39]. WEDM’s ability to achieve high precision and intricate designs has driven continuous advancements in wire properties, process optimization, and hybrid machining techniques.

**Figure 2:**
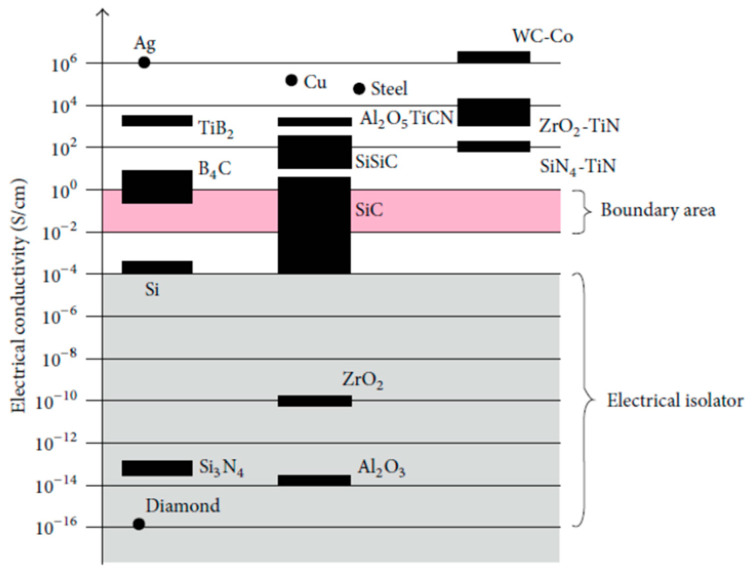
Electrical conductivity of different materials [25].

Despite these improvements, challenges such as tool wear, surface roughness, and machining time remain active areas of research [47]. For example, the relatively slow rate of material removal compared to conventional machining can lead to longer production times for large components. Another significant limitation of this process is the repetitive breakage of wires, particularly under high-tension conditions. To address this, innovations such as single wires capable of providing multiple discharges have been developed, which help reduce wire vibrations and lower production costs [24,25,26,48]. A minimum conductivity of approximately 10^−2^ Ω^−1^ cm^−1^ is required for successful EDM operations. While metallic materials inherently meet this threshold, ceramics often fall below it. Figure 2 shows that electrically conductive ceramics such as titanium nitride (TiN), titanium diboride (TiB_2_), and silicon-doped silicon carbide (SiSiC) possess sufficient conductivity to be machined effectively. By contrast, ceramics that are not conductive, such as zirconia (ZrO_2_), silicon nitride (Si_3_N_4_), and aluminum oxide (Al_2_O_3_), cannot be machined through EDM techniques without their conductivity being adjusted.

## 2. Material Removal Mechanisms in WEDM

**Figure 3:**
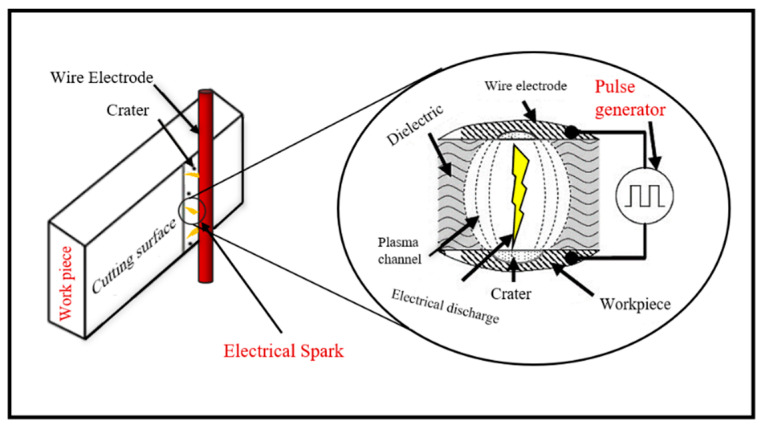
An overview of the spark erosion mechanism in the Wire EDM process.

Thermoelectric theory states that electrical power is converted into heat as a result of the electrical wire discharge during machining. This technique is considered unique in that, based on this theory, it relies on erosion effects to remove material, where electrical power is converted into heat energy. When the tool and workpiece are placed close together and immersed in an ionized medium, periodic electric discharges are generated between them. Figure 3 illustrates the material removal mechanism in Wire Electrical Discharge Machining (WEDM).

**Figure 4:**
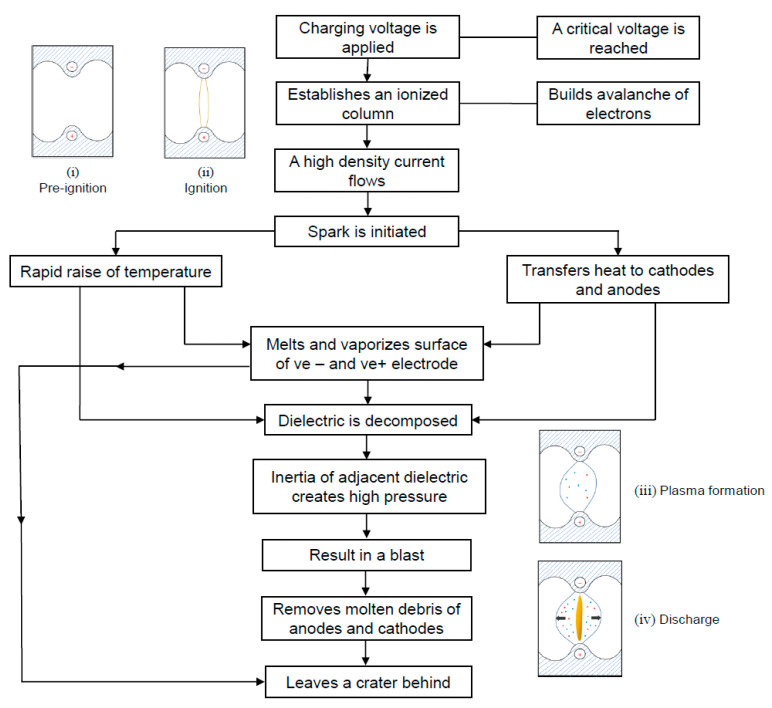
Stages of Wire EDM action.

An overview of the plasma cycle in Wire EDM is shown in Figure 4, outlining the stages of the process. Fluid acts as an insulator during the pre-ignition phase (Figure 4i), when no current flows through the tool and the workpiece. Two electrodes are then connected to a straightforward power source during the ignition phase (Figure 4ii), in which the circuit is closed to allow the electric current to flow, generating a voltage contrast in the middle of the tool and the workpiece. A line of molecules is formed in the middle of two electrodes, generating thousands of sparks per second. Electrons are discharged from the cathode, rush toward the anode, and crash into the dielectric fluid, where they split into electrons and positive ions due to the increased electrical potential between the two distinct electrodes. A plasma channel is formed by linked sparks between the cathode and anode (Figure 4iii), which causes thermal power at temperatures between 8000 and 20,000 °C that melts and vaporizes each pole’s material [49]. During the final stage of WEDM, the workpiece is continuously heated as ions and electrons continuously bombard the electrodes. A small portion of the workpiece material is eliminated as molten metal, which cools down, turns into debris, and subsequently removed from the discharge area by dielectric fluid. In the same way, craters develop on the surface of the workpiece, resulting in a rough machined look [50]. The amount of molten metal continues to increase as the plasma channel expands during this phase (Figure 4iv).

## 3. Process Parameters Influencing WEDM for SiC

**Figure 5:**
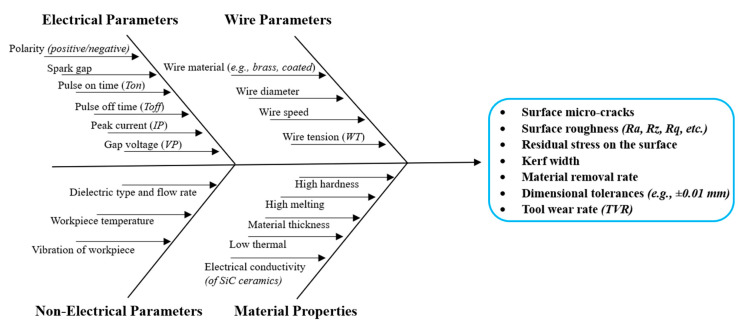
Cause-and-effect diagram for Wire EDM of SiC ceramics.

The Wire Electrical Discharge Machining process for silicon carbide ceramics is particularly affected by input machining operation variables such as wire material, tension, pulse duration, and machining current. In order to achieve precision, reduce defects, and maximize machining efficiency, careful selection of these parameters is essential. The most fundamental input parameters in the WEDM of SiC are pulse duration (T_on_), pulse-off time (T_off_), peak current (Ip), open voltage (OV), servo voltage (SV), wire speed, and wire tension. While the servo and open voltage regulate discharge stability and accuracy, the pulse duration and peak current impact thermal energy, which in turn affects material removal and crater sizes. Wire speed and tension secure stability in the wire, reducing vibrations and kerf width. The material removal rate, kerf width, surface roughness (Ra), machining rate, and thickness of the recast layer are all directly impacted by these factors. It is essential to select the optimal parameters for effective machining and to improve surface quality [51,52,53]. Figure 5 displays the cause-and-effect diagram for SiC ceramics in Wire EDM machining, highlighting how machining parameters affect performance and flaws such as surface microcracks, surface roughness, and kerf width. It is crucial to understand and enhance these elements to fully utilize WEDM’s potential for processing SiC in high-performance applications. Luis et al. [54] reported the successful EDM machining of siliconized/reaction-bonded silicon carbide (SiSiC). They found that material removal was primarily influenced by current strength and supplied voltage, whereas electrode wear was significantly affected by current strength, pulse timing, and plasma flushing efficiency. Additionally, Yamada et al. [55] found that EDM outperformed the diamond saw process in machinability, resulting in lower kerf loss. The machining rate increased with the wire electrode diameter, as larger wires achieved higher cutting speeds under consistent process parameters.

**Figure 6:**
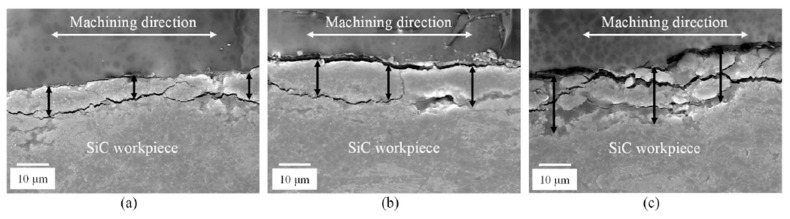
Comparison of the characteristics of recast layers with an SV of (**a**) 25, (**b**) 37, and (**c**) 51 V for an OV of 83 V. Reprinted with permission from Ref. [51]. Copyright 2020 Elsevier.

**Figure 7:**
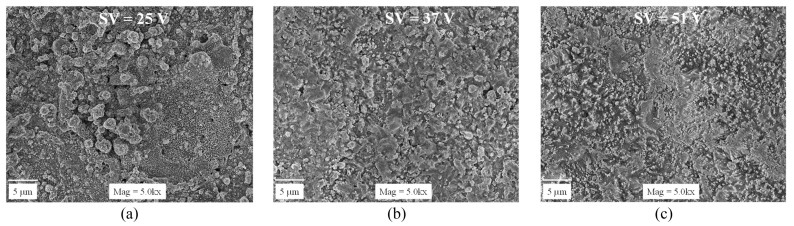
An analysis of the debris concentrations at an SV of (**a**) 25 (**b**) 37, and (**c**) 51 V. Reprinted with permission from Ref. [51]. Copyright 2020 Elsevier.

Singh et al. [51] mentioned that the most effective parameters influencing the WEDM of RHP-sintered SiC include the discharge energy, which increases the machining rate and efficiency; the duty factor, which slightly affects the machining rate but impacts debris removal and wire movement; and the servo voltage (SV), a dominant factor affecting discharge pulses and machining outcomes. A higher SV reduces debris concentration and minimizes material loss. Additionally, material removal is governed by melting–evaporation and thermal spalling, as indicated by the presence of recast layers and debris on the machined surface. Figure 6 illustrates the influence of servo voltage on the formation of recast layers, highlighting increased severity with higher SV due to greater thermal energy accumulation and transverse discharges. Figure 7 compares the debris concentration on machined surfaces at varying servo voltages (SV: 25, 37, and 51 V), illustrating the reduction in debris accumulation with increased SV due to enhanced flushing capabilities and reduced short-circuiting, which leads to better surface cleanliness at higher voltages. Pinargote et al. [56] reported the key process parameters that affect outcomes in WEDM of SiC-TiB2-TiC ceramic composites. For recast layer thickness (RLT), the pulse-on time and spark gap voltage were most significant, with increases in both these parameters increasing the RLT. For surface roughness (SR), spark frequency was the most influential, followed by the pulse-on time and spark gap voltage, with SR decreasing under optimized conditions, achieving a low surface roughness of 0.847 µm. The optimal parameter of U = 48 V, Ton = 1.0 µs, f = 10 kHz, q = 8 m/min yielded the thinnest RLT (3.16 µm) and lowest SR (0.847 µm), significantly reducing machining defects.

A key challenge in the EDM of ceramics is the requirement for a specially adapted pulse generator tailored to machining these materials. The pulse duration is a critical parameter influencing machining success, as it significantly impacts the development of the intrinsic conductive layer essential for non-conductive ceramics. Developing a control algorithm for precise pulse duration management is vital. Additionally, modeling the growth of this conductive layer could clarify its mechanism and reveal the effect of external factors or variables on operation [57]. Sánchez et al. [58] investigated and optimized die-sinking EDM and Wire EDM processes for machining boron carbide (B4C) and silicon-infiltrated silicon carbide (SiSiC). Their findings revealed that Wire EDM achieved surface roughness values as low as 0.56 µm for B4C and 3.5 µm for SiSiC. For die-sinking EDM, material removal rates (MRRs) of 8.3 mm^3^/min for B4C and 10.1 mm^3^/min for SiSiC were achieved. Furthermore, they demonstrated that a surface roughness below 1 µm could be obtained in die-sinking EDM if no capacitor was utilized in the process. Table 4 offers a comprehensive overview of investigations into Wire EDM of carbide ceramics, including the specific machining parameters, precise input variables, and corresponding outcomes.

## 4. Electrode Selection in WEDM Technology

In the WEDM process, the chosen electrode material mainly depends on the tensile strength and required proficiency. The most common electrode materials utilized in Wire EDM technology are brass, copper, and molybdenum, as well as materials coated with zinc and tungsten. Selecting the appropriate electrode material for each application is essential to achieve higher efficiency in the machining process, a high-quality processed surface, and a reduction in electrode wear. The polarity and shape of the electrode can control its lifespan during semiconductor processing. The main factors determining electrode wear in Wire EDM are the process temperature and wire size [51,66,67]. It is inevitable that electrodes will wear out from exposure to high-temperature plasma. Occasionally, semiconductor materials are used to modify electrodes’ electrical features through introducing impurities into pure intrinsic semiconductors. Semiconductors are categorized into two types according to the dopants used: P-type semiconductors, which use boron as a dopant, and N-type semiconductors, which use phosphorus. When the polarity of the electrode aligns properly with the dopant of the workpiece, the MRR, electrode loss, and quality of the surface can be reduced. Zhao et al. [48] evaluated the influence of various tool polarities on discharge current, material removal rate (MRR), and electrode wear under machining operation on silicon carbide. In the findings, negative polarities are best suited to short-pulse-duration machining. Specifically, negative polarity led to a higher material removal rate of 0.12 mm^3^/min in comparison to 0.08 mm^3^/min for the positive polarity, as well as a lower tool wear rate. The authors also emphasized that this phenomenon is the main reason for the high material removal rate in SiC material because of its high brittleness. Reynaerts et al. [68] found that galvanized brass wire with different dopants can be a practical choice in the machining of silicon. Negative and positive electrodes were used on P-type and N-type silicon, respectively, to overcome potential interface resistance. This study showed that a high semiconductor resistance impacts the feed rate and material removal rate (MRR), but not the surface integrity. Moreover, by controlling the wire vibration factor and applying optimized machining parameters, an impressive enhancement in slicing speeds of about 40–50% was achieved, accompanied by a considerable decrease in cutting losses of 20% [69]. Table 5 shows commonly used wire materials in Wire EDM technology.

## 5. Advances in WEDM Technology for SiC

**Figure 8:**
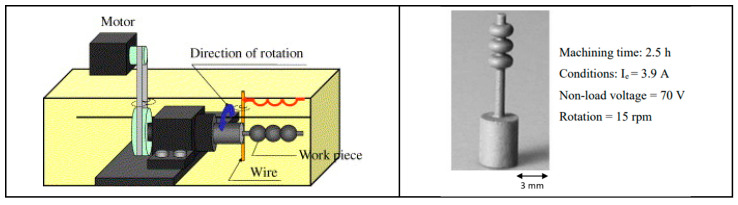
Cutting of small silicon nitride products using Wire EDM. Reprinted with permission from Ref. [59]. Copyright 2004 Elsevier.

The distinctive features of silicon carbide (SiC), such as its hardness and brittleness, can cause considerable challenges for machining. Conventional machining methods often lead to high material loss and surface damage, highlighting the necessity for creative techniques. Wire Electrical Discharge Machining (WEDM) has become a favorable option because of its non-contact characteristics, which reduce mechanical stress. Despite its potential, there are challenges in machining SiC using WEDM, such as variations in kerf width, ineffective processing, and wire instability. Electrical conductivity is a vital factor in making WEDM suitable for SiC ceramics, which are naturally low-conductivity, potentially limiting their machinability. A significant advancement has been made by integrating conductive layers such as titanium nitride (TiN) and borides such as TiB2 into the ceramic matrix. These adjustments improve the electrical properties of SiC, allowing stable discharge conditions during WEDM and making it easy to attain precision machining [47,79,80,81,82,83]. Figure 8 demonstrates the machining of the axisymmetric rotational workpiece (silicon nitride) through the WEDM technique. A. Kimura’s research signifies a notable step forward in addressing these challenges through the development of a multi-wire EDM slicing method. This strategy tackled problems such as the inconsistent wafer thickness and high kerf loss generally connected with conventional multi-wire saw techniques [84]. The main innovation was the use of the track-shaped brass-coated steel wire electrode, which enhances wire tension and also stability. The experimental findings showed that fixture plate support and optimized traverser configurations can effectively control wire vibrations, which can lead to improved slicing quality and reduced kerf width. Ogawa et al. [85] presented a new pulse control system designed to reduce arcing and avoid wire breakage during the simultaneous machining of multiple SiC wafers in the multi-wire EDM process. They mentioned that a rise in discharges along the transverse direction with deeper machining results in remarkable variations in wafer thickness across the surface. A comparison between cryogenic EDM with liquid nitrogen and conventional EDM, conducted by Srivastava and Pandey [86], shows significant improvements in machining performance, with liquid nitrogen reducing tool wear by up to 20% and surface roughness by as much as 27%. Furthermore, the cryogenic technique enhances tool shape retention during machining, as evidenced by superior roundness profiles. As shown by these results, liquid nitrogen can minimize wear and improve surface quality, making it a promising technique for advanced machining.

**Figure 9:**
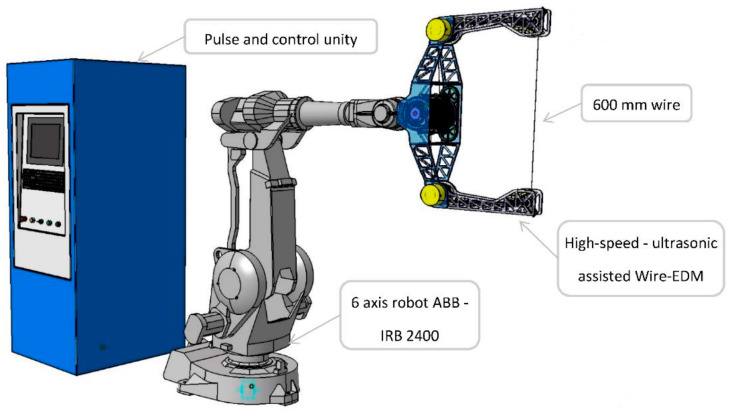
Schematic of high-speed ultrasonic-assisted WEDM robot [88].

Post-WEDM treatments have emerged as a critical advancement to address surface damage and improve the mechanical performance of machined SiC ceramics. Techniques such as ultrasonic machining and abrasive blasting are particularly effective in restoring surface integrity, reducing roughness, and enhancing material properties such as hardness and strength. Research has shown that these finishing techniques can also significantly improve the Weibull modulus, which is a measure of the reliability and consistency of the material. For example, Deng et al. [87] illustrated that ceramics machined with EDM had reduced hardness and increased surface roughness, although subsequent treatments reversed this. Ultrasonic machining in particular provided superior results, increasing the Weibull modulus and mechanical strength. Almeida et al. [88] suggested a force-free, flexible machining solution for exotic materials by incorporating Wire EDM with six-axis robotic arms, as can be seen in Figure 9. This approach aims to tackle the limitations of CNC machining and the inaccuracies of robotic machining by using innovative end-effector design and the application of the TRIZ method, even with current technological constraints.

Beyond surface roughness, the integrity of the subsurface is a critical concern in the WEDM of SiC. The intense, localized heat from the spark erosion process leads to the formation of a ‘recast layer’ on the machined surface. This layer is composed of molten and resolidified material, often mixed with debris from the wire electrode and byproducts from the dielectric fluid. For brittle ceramics like SiC, this thermal cycling can induce significant subsurface damage, including microcracks that propagate beneath the recast layer. Furthermore, the rapid quenching of the molten material can lead to the formation of an amorphous phase within the recast layer, altering the material’s mechanical and electrical properties. Surface oxidation is another common phenomenon, where the high temperatures promote a chemical reaction between the SiC workpiece and the oxygen present in the dielectric fluid, forming a silica (SiO_2_) layer. These thermally induced effects can compromise the component’s flexural strength and reliability. Therefore, post-processing techniques such as ultrasonic machining and abrasive blasting are often essential to remove this damaged layer and restore the desired surface integrity.

### 5.1. Cryogenic Wire EDM: Enhancing Efficiency and Practicality

**Figure 10:**
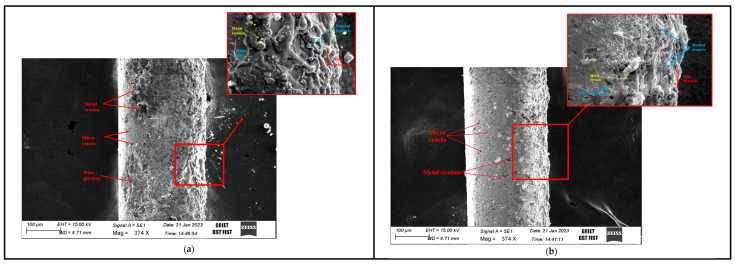
Machined surfaces with different electrodes: (**a**) untreated electrode; (**b**) treated electrode [96].

To enhance the machinability of hardened materials, a process known as cryogenic treatment is applied to cutting tools before machining operations. This treatment involves subjecting the tools to extremely low temperatures, typically utilizing liquid nitrogen, which helps transform the microstructure of the tool materials. In cryogenic treatment, materials are kept at very low temperatures for a specific duration, resulting in improved electrical properties that are particularly important in electric discharge machining [89]. Electrodes with superior electrical properties can remove material at high speeds and provide a smooth surface finish [90]. In addition, the life and wear resistance of the cutting tools are also enhanced by suitable cryogenic treatment [91]. In this treatment, the cooling time, soaking rate, and temperature are critical parameters that alter the properties of alloys [92,93,94]. In order to achieve the desired material properties, researchers have optimized these parameters. Three specific sub-zero temperature ranges are primarily used for cryogenic treatment: (i) 223 to 193 K (cold/cryogenic treatment), (ii) 193 to 113 K (shallow cryogenic treatment), and (iii) 113 to 77 K (deep cryogenic treatment) [95]. Kosaraju et al. [96] carried out a study centering around optimizing WEDM parameters, specifically the pulse-off time, finding that employing cryogenically treated zinc electrodes notably enhances material removal, surface quality, and microstructural factors when machining Inconel 600, presenting strengthened bonding, reduced recast depth, and minimized electrode erosion. Figure 8 shows that the electrode wear rate has a direct impact on recast depth, MRR, and Ra. Electrodes that have not been cryogenically treated (Figure 10a) exhibit more microcracks and pitching, while those that have been cryogenically treated (Figure 10b) exhibit finer grains, uniform orientation, and reduced erosion, which enhance surface integrity and reduce wear. Table 6 presents a list of the most common cryogenic fluids along with detailed information about each.

Although cryogenic treatment has been extensively studied in various machining applications, its potential in Wire EDM for SiC ceramics remains underexplored. Using cryogenic techniques in Wire EDM could help enhance electrode performance and potentially minimize thermal damage during machining. Additionally, cryogenic treatment enhances the wear resistance and lifespan of tools, leading to more cost-effective and sustainable machining solutions. By optimizing this approach, researchers could discover new methods to enhance the material removal rates, surface quality, and overall machining efficiency of SiC ceramics. Consequently, this topic warrants further investigation as a potentially transformative contribution to advanced manufacturing technologies.

### 5.2. Eco-Friendly Approaches and Challenges in WEDM

SiC ceramics demonstrate distinct sustainability challenges because of their low electrical conductivity, extreme hardness, and abrasive wear behavior. These aspects can cause higher wire wear and longer processing times and accelerate dielectric degradation, thereby amplifying the environmental impact of the WEDM process. On the other hand, the increasing focus on sustainable manufacturing has sparked substantial interest in eco-friendly methods within Wire Electrical Discharge Machining (WEDM). Gas emissions from hydrocarbon fluid are unavoidable in conventional EMD/WEDM processes, and although Wire EDM (WEDM) produces fewer fumes compared to die-sinking EDM, exposure to hydrocarbon-based dielectrics can present health risks, including skin irritation. Moreover, there are concerns about air quality due to the release of electromagnetic radiation [97], as well as about energy consumption, as WEDM requires continuous electrical discharges, resulting in substantial electricity usage [98]. This leads to increased production costs and a larger carbon footprint, with rising energy prices and global CO_2_ emissions heightening the emphasis on sustainable manufacturing [99]. Approximately 90% of energy used in industry is related to product manufacturing [100], and 75% of that electricity is used by machine tools [101]. Therefore, it is essential to optimize the energy consumption of Wire EDM machines. It is especially important to take these concerns into account when working with SiC ceramics, which typically require longer machining times because of their poor electrical conductivity and high material hardness.

A direct comparison of energy efficiency with other techniques is complex; however, while WEDM is an energy-intensive process due to the thermoelectric removal mechanism, its ability to machine near-net-shape parts can reduce material waste and the need for subsequent energy-intensive finishing steps common to conventional grinding, which is an important consideration for its overall life-cycle energy footprint.

Negrete et al. [102] highlighted the remarkable progress in sustainable manufacturing through optimizing WEDM processes. They used a multi-objective optimization strategy that consists of Taguchi design and a desirability analysis to attain outstanding decreases in energy use and CO_2_ emissions. Specifically, there was a reduction in energy consumption by up to 14%, and CO_2_ emissions decreased by 14.09% for thicker workpieces. This was mainly due to the pulse-on time, which was identified as the most significant factor affecting energy efficiency. Furthermore, the machining duration decreased by 16%, bringing about an enhancement in production efficiency and a reduction in operational costs. The results draw attention to the potential of optimizing process parameters to create more sustainable WEDM operations, indicating a shift toward eco-friendly manufacturing methods with improved energy efficiency, lower emissions, and reduced costs. Sampath et al. [103] presented remarkable advancements in sustainable manufacturing through the implementation of a near-dry WEDM system that uses a combination of compressed air and water as its dielectric fluid. This eco-friendly strategy decreases dependence on hydrocarbon-based dielectrics, minimizing harmful gas emissions and health risks for operators. The study showed that optimizing key factors, such as air pressure and water flow rate, led to reductions in gas emission concentration (GEC) and relative emission rate (RER) by up to 32.03 and 85.01%, respectively. Moreover, an improved material removal rate (MRR) was also noted, demonstrating that sustainable machining can be achieved without sacrificing production efficiency. These findings point to the ability of near-dry WEDM to promote green manufacturing through reduced environmental effects, increased workplace safety, and supporting the transition to more environmentally friendly production methods. Despite recent developments in green WEDM techniques, their direct application to SiC ceramics remains unexplored. Future work should concentrate on adapting these strategies to address the specific sustainability challenges presented by ceramic machining.

Table 7 provides an overview of recent eco-friendly WEDM approaches that emphasize sustainable strategies and process optimizations and substitute dielectrics designed to minimize emissions and energy use. Although many studies focused on metal or composite materials, these methods present a promising direction for improving the sustainability of SiC machining.

While these eco-friendly approaches show promise, a comprehensive Life-Cycle Analysis (LCA) of WEDM for SiC ceramics has not yet been conducted. Such an analysis would need to quantify the environmental impact across the entire process chain—from the energy-intensive production of SiC material and dielectric fluids to the operational energy consumption, waste management of degraded fluids and wire, and aerosol emissions. A formal LCA would be a valuable direction for future research, enabling a holistic comparison of WEDM against other advanced ceramic machining techniques from a sustainability point of view.

## 6. Conclusions

This review discusses the current state of Wire Electrical Discharge Machining (WEDM) for silicon carbide (SiC) ceramics, highlighting its ability to overcome the challenges of machining hard and brittle materials. Future research on WEDM technology for silicon carbide ceramics should address several key challenges. SiC’s very high melting point makes it difficult to erode effectively using electrical discharges, resulting in slower cutting speeds and reduced efficiency. Additionally, SiC’s high thermal conductivity dissipates the heat generated during the WEDM process, reducing the effectiveness of the cutting action and leading to inconsistent performance and increased wear on the wire electrode. The hardness of SiC can cause significant wire vibration and breakage, affecting cutting accuracy and overall efficiency. Achieving a high-quality surface finish on SiC using WEDM is also challenging, as the process may result in surface roughness and microcracks due to intense thermal and mechanical stresses. To overcome these challenges, several potential technological solutions can be explored:

- Using assisting electrodes can enhance the electrical conductivity of SiC during the WEDM process by introducing a conductive layer or material that is in contact with the SiC workpiece.
- Adjusting the pulse duration, discharge energy, and frequency can improve the efficiency of WEDM for SiC, with shorter pulse durations and lower discharge energies reducing thermal damage and improving surface quality.
- Utilizing advanced wire materials, such as coated or composite wires, can reduce wire wear and breakage, while specialized dielectric fluids with enhanced cooling and flushing capabilities can help dissipate heat more effectively and remove debris from the cutting zone.
- Implementing multi-wire EDM techniques can increase cutting efficiency and reduce kerf loss, as multiple wires are used simultaneously to slice through the SiC material.
- Combining WEDM with other machining processes, such as laser or ultrasonic machining, can enhance overall cutting performance. For instance, laser-assisted WEDM can be used to pre-treat the SiC surface, creating controlled microcracks that facilitate increased material erosion via subsequent electrical discharges. Similarly, ultrasonic-assisted WEDM, where high-frequency vibrations are applied to the wire or workpiece, can improve debris evacuation and dielectric circulation in the cutting zone, leading to a more stable process and improved surface finish. These hybrid approaches synergistically combine the strengths of different processes to overcome the inherent challenges of machining SiC. Finally, advanced temperature-control systems can help manage the heat generated during the WEDM process, ensuring consistent and high-quality cuts.
- To bridge the gap between academic research and industrial application, collaborative efforts are needed to establish standardized WEDM process parameters and testing protocols for different variants of SiC ceramics. This would enhance process reliability and consistency, a crucial step for wider adoption.
- Recent advancements in manufacturing are increasingly leveraging Artificial Intelligence (AI) and Machine Learning (ML) to optimize complex processes, and WEDM is no exception. For SiC machining, where the process window for achieving high quality and efficiency is narrow, AI offers significant potential. As noted in the literature, models such as Artificial Neural Networks (ANNs) and Machine Learning algorithms are already being used to predict outcomes for other materials. Applying these techniques to SiC could involve developing models to predict surface roughness, material removal rate, and the likelihood of microcrack formation based on real-time sensor data (e.g., discharge voltage, acoustic emissions). Such intelligent systems could enable real-time parameter adjustments to maintain process stability, prevent wire breakage, and ensure consistent part quality, moving beyond static optimization tables toward adaptive, intelligent manufacturing.

## Figures and Tables

**Table 1:** High-performance applications of SiC across various industries, highlighting its properties.

Industry	Part		Description
Power	Used in power devices such as MOSFETs and diodes [2]	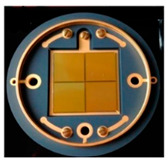 [3]	These components are usually small, measuring in the millimeter range, which allows for compact and efficient power conversion systems.
Defense	Armor [4]	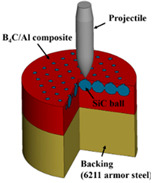 [5]	Large silicon carbide (SiC) components, measuring from centimeters to meters, are utilized for their exceptional strength and ability to withstand extreme conditions.
Aerospace	Gas turbine engines	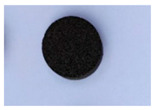 Reprinted with permission from Ref. [6]. Copyright 2022 Elsevier.	These components experience extremely high temperatures and harsh conditions within the engine. SiC’s exceptional thermal stability, high strength, and resistance to oxidation and thermal shock make it an ideal material for these applications.
	Nozzle vanes	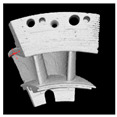 [7]	These components must possess thermal stability and oxidation resistance under extreme temperature conditions.
Automotive	(EV) powertrains	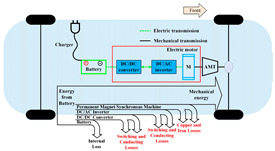 [8]	The size of these components can vary, but they are generally designed to fit within the compact spaces of automotive systems.
Biomedical devices	Biosensors [9], neural implants [10], dental implants [11]	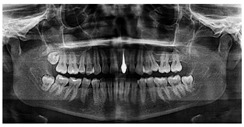 [12]	Small components, ranging from millimeters to centimeters, are utilized for their biocompatibility and durability in advanced medical applications.
Industry	Bearings	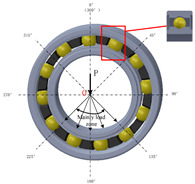 [13]	SiC is used in the production of high-performance brake disks. These discs offer superior thermal conductivity and wear resistance, improving braking performance and durability.
	Mechanical seals	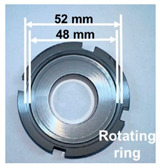 [14]	SiC is used in mechanical seals for pumps and compressors, providing excellent wear resistance and extending the lifespan of these components.
	Cutting tools	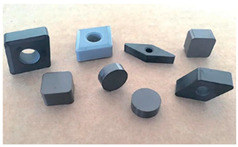 [15]	These are ideal for machining hard materials due to their exceptional wear resistance and hardness, making them suitable for cutting and grinding applications.

**Table 2:** Key properties of silicon carbide (SiC).

Property	Value	Description
Density	~3.15–3.21 g/cm^3^	Low density contributes to lightweight components in high-performance applications.
Hardness	~25–28 GPa (Vickers)	Extremely high hardness, making it one of the hardest ceramics available.
Thermal Conductivity	~120–180 W/m·K	Excellent thermal conductivity, ideal for high-temperature environments.
Thermal Expansion	~4.0 × 10^−6^/K (20–1000 °C)	Low thermal expansion ensures dimensional stability under temperature variations.
Flexural Strength	~350–500 MPa	High strength across a wide range of temperatures.
Compressive Strength	~3900 MPa	Exceptional resistance to compression, ideal for structural applications.
Elastic Modulus	~410 GPa	High stiffness is suitable for precision engineering applications.
Electrical Conductivity	Semi-conductive	Conductivity varies depending on doping; used in electronic devices.
Chemical Resistance	High	Resistant to oxidation, acids, and bases, enhancing longevity in harsh environments.

**Table 3:** Properties of SiC variants and implications for WEDM.

Property	SSiC	SiSiC	Doped SiC
Electrical Conductivity	Very low	Moderate (due to Si)	Moderate to high
Hardness (Vickers, GPa)	~25–28	~22–26	~20–27
Thermal Conductivity (W/m·K)	~120–180	~130–160	~140–180
Brittleness	High	Moderate	Moderate
WEDM Suitability	Requires enhancement	Suitable	Suitable

**Table 4:** Input variables and outcomes explored in various studies on Wire EDM of carbide ceramics.

Reference	Electrode	Assisting Electrode	Polarity	Peak Current	Pulse Duration	Pulse-off Time	Interval Time	Open Voltage	Servo Voltage	Wire Tension	Wire Feed Rate	Dielectric Flushing Pressure	Outcomes
[59]	Brass (Ø 0.2 mm)	TiN (t = 3 μm)	Negative	3.9 –4.9 A	4 μs		16 μs			2.45 N			They reported that the most important considerations for slicing ultra-thin wafers are balancing the wafer thickness, kerf loss, and slicing rate. A wafer thickness of 150 μm with a slicing rate of 1.05 mm/min and kerf loss of 121 μm is optimal for minimizing material waste while maintaining efficiency. The use of response methodology and composite desirability optimization reduced kerf loss by 40–50%. Surface analysis revealed crack-free wafers with minor defects such as dents and holes, highlighting complex material removal phenomena during machining.
[60]					0.3 μs	20 μs				4 N	62	10 (bar/kg)	By increasing servo voltage, the kerf loss and the inter-electrode gap also increased, leading to significant debris accumulation on the machined surface.
[25]	Brass (Ø 0.2 mm)		Negative					80 V	58 V		8	8	When the tool electrode has a negative polarity, a higher machining speed and a lower tool wear ratio will be reached during foil EDM of SiC with short pulse durations. Due to the larger kerf width and slower machining speed, long pulse durations are not acceptable for SiC EDM.
[61]	Brass (Ø 0.2 mm)	50.85 mm/14.3 mm		1	4 μs	3.5 μs		100 V	75 V	20 N	80 m/min	2 l/min	They investigated the efficiency and quality of slicing under oil- and water-based Wire Electrical Discharge Machining (WEDM) conditions. Water machining is faster at a discharge current of 1 A, but only by about 1.1-fold compared to that of oil machining. Additionally, the average width of the machined groove during water machining at 1 A is smaller than that of oil machining. The groove width remains wider even at a discharge current of 0.7 A for water machining compared to a discharge current of 1 A for oil machining.
[62]	Zinc-coated (Ø 0.25 mm)		Negative	11.2 A		12.82 (μs)			42 V	600 g	10 m/min		Because of its high electrical resistivity and thermal conductivity, SiSiC requires a high sparking power during EDM machining. To initiate the spark and for it to remain stable, in this study, a high peak current was necessary. The high thermal conductivity of the material triggered heat loss owing to the conductivity that decreased the energy output of the operation. The connection between machining parameters and several responses was established experimentally. Statistical analysis provided a clear understanding on the influence of the machining parameter, elucidated by validated mathematical models.
[63]	(EDM-C3 grade)		Negative	32 A	3.2 µs		100 µs	−200 V					They observed that using high-energy parameters, such as elevated peak currents and prolonged discharge durations, during the machining of conductive SiC led to instability in the process and caused significant damage to both the surface and subsurface of the material. When material melts and evaporates with high-energy discharge, there is not enough time for it to be ejected before it resolidifies.
[64]	Molybdenum (Ø 0.18 mm)		Workpiece (+)	6 A	5 µs, 10 µs, 20 µs, 40 µs, 80 µs.	8 × T_on_	T_on_ + T_off_	140 V			7.2 m/s		They assessed the damage of a particular crystal face of single-crystal silicon, studying various input variables for EDM. In experiments with a variable pulse width, differences were found in material removal mechanisms. Analysis indicates that, during semiconductor processing, the melting and vaporization resulting from prolonged discharge pulses do not remove more material than the thermal stresses and explosive forces generated by brief pulses. Extended pulses often result in a concentration of the discharge, which can lead to an unpredictable process.
[65]	Molybdenum (Ø 0.18 mm)			2 A, 3 A, 4 A.	100 µs, 130 µs, 150 µs.						11 m/min	30 kPa, 40 kPa, 50 kPa.	In the examination of material removal rate (MRR), it was discovered that the pulse-on time, along with its interplay with powder concentration and current, was an important factor. Pressure also proved to be significant. The findings demonstrated that a longer pulse-on time was associated with a lower MRR. Furthermore, the outcomes were exactly understood when these relations were taken into account. Specifically, a higher pulse-on time combined with low to average powder concentrations (2 and 4 g/l) is expected to distribute or dilute the effect of heat intensity, which in turn reduces the MRR. However, increasing the powder concentration to 6 g/l resulted in an improvement, likely due to enhanced bridging phenomena.

**Table 5:** Typically used wire materials in Wire EDM technology.

Author/Year	Wire Material	Work Material	Input Variables	Responses	Optimization Method	Key Observations
[70] Sheth et al. (2020)	Molybdenum (Ø 0.018 mm)	Mg-Zn-RE-Zr Alloy	Current, Ton, Toff, Wire Feed Rate	Surface Roughness	CCD + RSM + PVS	Toff is least influential; Ton and current are dominant
[71] Ali et al. (2025)	Molybdenum (Ø 0.18 mm)	AA2024/Al_2_O_3_/SiC/Si_3_N_4_/BN Hybrid Composite	Voltage, Current, Gap, Wire Feed, Wire Speed	Ra, CS, KW	Taguchi DOE, Desirability Index, SEM, Topography, ANOVA	Achieved smoother Ra and higher CS for complex profiles using optimal WEDM settings; reduced KW and minimized craters
[72] Das & Joshi (2025)	Zinc-Coated Brass (Ø 0.25 mm)	Ti-6Al-4V	Voltage, Current, Pulse-On/Off Time	Crater Volume, Wire Stress, Wire Failure Risk	3D Thermo-Mechanical FEM, Experimental Validation	Predicted wire breakage based on crater volume; defined wire failure threshold; model improves tool longevity and sustainability
[73] Soundararajan et al. (2020)	Zinc-Coated Brass (Ø 0.25 mm)	A413 + 12 wt% B_4_C Composites	Ton, Toff, Peak Current	MRR, Surface Roughness	CCD + RSM	Ton significantly affects MRR and SR; Toff reduces SR
[74] Kumar et al. (2019)	Brass (Ø 0.25 mm)	Inconel 825	Ton, Toff, Peak Current, Spark Gap, Wire Tension, Wire Feed	MRR, Surface Roughness, Wire Wear Ratio	RSM + Desirability Approach	Ton, gap voltage, and peak current positively impact MRR; Toff negatively affects SR
[75] Alduroobi et al. (2020)	Brass (Ø 0.25 mm)	AISI 1045 Steel	Ton, Toff, Servo Feed (SF)	MRR, SR	ANN	MRR optimal: Ton = 25 μs, Toff = 20 μs, SF = 30 mm/min; SR optimal: Ton = 10 μs, Toff = 40 μs, SF = 0 mm/min
[76] Abbas et al. (2023)	SS-304, Copper, Brass	Al/SiC/Gr Composite	Ton, Toff, SV, I, Tool Electrode	MRR, TWR	RSM + COPRAS + Machine Learning	Ton: 60, Toff: 60, SV: 7, I: 12; brass tool had highest TWR
[77] Sharma et al. (2013)	Brass	High-Strength Low-Alloy Steel (HSLA)	Ton, Toff, Peak Current, SV	MRR, Surface Roughness	RSM	Ton ↑ → SR ↑; Toff ↑ & I ↓ → better SR
[78] Khanna et al. (2022)	Brass (Ø 0.25 mm)	Al/SiC/Ti Hybrid Composite	Ton, Toff, SV, Wire Feed	Cutting Speed (CS), Kerf Width (Kw)	BBD + RSM + Teaching-Learning-Based Optimization (TLBO)	Ton strongly affects CS; SV influences Kw

These arrows (↑, ↓) show how increasing or decreasing affects each parameter in the study, indicating whether it increased or dropped.

**Table 6:** Cryogenic fluids, their temperature, and availability in nature.

Cryogenic Fluid	Temperature (°C)	Nature	Availability in Nature	Notes
Helium 3	−269.96	Inert Gas	5 ppm	Rare isotope
Helium 4	−268.94	Inert Gas	5 ppm	Common isotope
Hydrogen	−252.88	Combustible	0.6 ppm	Highly combustible gas
Neon	−246.06	Neutral	18 ppm	Used in lighting
Nitrogen	−196.06	Neutral	78.09%	Major component of air
Mixture of Air	−194.35	Neutral	100%	Air composition
Fluorine	−187.91	Toxic	0.6 ppb	Highly toxic
Argon	−185.91	Inert Gas	0.94%	Used in welding
Oxygen	−182.97	Flammable	20.95%	Supports combustion
Methane	−161.45	Flammable	1866 ppb	Natural gas component
Carbon Dioxide	−56.6	Neutral	0.04%	Greenhouse gas

**Table 7:** List from literature review.

Author(s), Year	Eco-Friendly Approach	Method/Principle	Environmental Benefit	Relevance to SiC Machining
[104] Boopathi et al., 2021	Near-Dry WEDM	Compressed air + water mist as dielectric	Reduces toxic emissions, improves operator safety	The possibility of reducing dielectric contamination in brittle ceramics such as SiC
[104] Boopathi et al., 2021	Pulse Parameter Optimization	Adjusting pulse-on/off times and discharge energy	Reduces energy usage and gas emissions	Adjusting for SiC conductivity limits may improve both efficiency and quality
[105] Das et al., 2020; [102] Camposeco-Negrete., 2021	Green Dielectrics (e.g., Bio-Oils)	Use of biodegradable dielectrics like vegetable oils or nano-emulsions	Lower toxicity, safer handling, reduced disposal issues	Under investigation for ceramics such as SiC
[106] Ng et al., 2016	Multi-Objective Optimization (Taguchi + TLBO)	Hybrid optimization method for energy, Ra, MRR, and kerf width	Balances multiple eco-efficiency metrics	Can be adapted for SiC if mechanical and electrical properties are considered
[107] Venkatarao et al., 2023	Deionized Water as Dielectric	Substitution of hydrocarbon oils with deionized water	Lower environmental impact, cleaner machining process	Effective for SiC when combined with auxiliary electrode or conductivity coating
[108] Ming et al., 2019	Magnetic-Field-Assisted WEDM	Enhancing debris evacuation using magnetic fields	Improves MRR and reduces thermal input	Underexplored for SiC, but promising for heat-sensitive materials
[109] He et al., 2020	Energy Prediction Using ML	Machine learning-based prediction of energy consumption	Enables pre-optimization of energy-intensive cycles	Could guide parameter design in SiC machining for better efficiency

## Data Availability

No new data were created or analyzed in this study. Data sharing is not applicable to this article.
